# The burden of tooth loss in Italian elderly population living in nursing homes

**DOI:** 10.1186/s12877-018-0760-0

**Published:** 2018-03-20

**Authors:** Fabio Cocco, Guglielmo Campus, Laura Strohmenger, Viviana Cortesi Ardizzone, Maria Grazia Cagetti

**Affiliations:** 1 Department of Surgery, Microsurgery and Medical Sciences, School of Dentistry, University of Sassari, Viale San Pietro 43/C, I-07100 Sassari, Italy; 2 WHO Collaborating Centre for Epidemiology and Community Dentistry, University of Milan, Milan, Italy; 3 Department of Biomedical, Surgical and Dental Sciences, University of Milan, Milan, Italy

**Keywords:** Edentulism, Cognition, Epidemiology, Nutrition

## Abstract

**Background:**

This survey aims to evaluate the prevalence and severity of tooth loss in the Italian elderly population living in nursing homes and to associate the oral data with demographic, socioeconomic factors, the Mini-Mental State Examination (MMSE), the Body Mass Index (BMI) and the Mini Nutritional Assessment (MNA) scores.

**Methods:**

A cluster sample method was performed using each nursing home as a cluster. Twenty-three nursing homes located in the five areas of the Italy (North-West, North-East, Centre, South and Islands) were selected. An informed consent to participate was distributed by the personnel of the selected nursing homes and signed directly by subjects/caregivers; 2114 forms were distributed, 1998 forms signed and finally 1976 subjects were examined. Chewing ability was scored as good (≥10 functional units (FUs)), sufficient (7–10 FUs), and insufficient (< 7 FUs). The presence of prosthetic dental restorations was summarized as: absent of prosthesis, fixed prosthesis, removable prosthesis, combined prosthesis. Age, gender, socioeconomic status, MMSE, BMI and MNA were obtained from medical charts.

**Results:**

Almost three quarters of the subjects were ≥ 80 years old (74.37%) and women (74.04%). The prevalence of edentulism was 42.10% with a large variation among the five areas of Italy (from 34.43% in Centre to 53.46% in North-West). Insufficient presence of FUs was preeminent in each age group (prevalence 42.10%) and statistically associated to age and to female gender (*p* <  0.01). Overweight/obese (7.47%) subjects showed the highest FUs. Area of living, MMSE (both < 0.01), BMI (*p* = 0.01) were statistically significant associated to the type of prosthetic dental restorations in the oldest group. Subjects with no mental impairment showed the highest percentage of prosthetic dental restorations (32.36%).

**Conclusions:**

More than half of the sample has an insufficient number of functional units for chewing and this is more pronounced in females. The presence and the type of prosthetic dental restorations are linked to cognitive impairment: the higher is the mental impairment the higher is the number of subjects with absence of prosthetic restorations. The findings of this national survey highlight the need for public health policy, aiming to increase awareness regarding oral health though health education.

**Electronic supplementary material:**

The online version of this article (10.1186/s12877-018-0760-0) contains supplementary material, which is available to authorized users.

## Background

Italy, as several industrialized and BRICST (Brazil, Russia, India, China, South Africa and Turkey) countries around the world, has been experiencing an epidemiological transition: a reduction in birth rates and an increase in life expectancy, resulting in an enlarged elderly population [1]. In Italy on January 1st 2016, 22.04% of residents were above 65 years of age, 6.67% of which were older than 80 years. The prevalence of sexagenarians and octogenarians has increased over time, from 6.2% and 0.7% in 1901 to 20.1% and 5.6% in 2009. In 2016, the average life expectancy at birth in Italy was 82.7 years, one of the highest in the world [2]. These demographic changes clearly have important public health implications [3]. The report from the Global Burden of Disease 2010 Study showed that as the population’s average age increases, oral health issues also increase [4, 5]. The stomatognathic system has important functions in each stage of the individual’s life; the presence of a functional dentition is essential also for the quality of life in elderly age [6, 7]. WHO indicates that the number of remaining teeth is an essential data for oral health surveillance [8], encouraging the evaluation of risk factors related to tooth loss in the elderly population and its impact on life [8], issues not tackled sufficiently in literature.

Edentulism, defined as the loss of all teeth, is a risk factor for malnutrition [9, 10]. Although in the last three decades the prevalence of edentulism has decreased in industrialized countries, a large fraction of elderly is still edentulous (almost three millions in the UK). Severe tooth loss (presence of less than seven teeth) is ranked as the 26th most prevalent condition in the Global Burden of Disease 2010 Study, with an estimated global prevalence of 2%. Differences in geographical areas seem to influence this condition [10, 11]. Even if the presence of a shortened dental arch (premolar to premolar) is considered to provide acceptable chewing function, a higher number of teeth contributes in providing better general health conditions.

Poor oral health habits have been associated with higher caries and periodontal diseases prevalence, which, in turn, increases the risk of tooth loss [12]. The frequency of dental care and the availability of dental services may affect the number of remaining teeth in the later stages of life [13, 14].

In Italy about 3% of adults over sixty years of age lives in nursing homes but a large variability among the different areas of the country is reported [15].

Despite the high percentage of elders living in Italy, only one local survey has been performed describing the prevalence of tooth loss amongst this population [9]. The collection of this data is a prejudicial factor in establishing public health policies to prevent or treat this condition.

This survey aims to evaluate the prevalence and severity of tooth loss in the Italian elderly population living in nursing homes and to associate the oral data collected with demographic, socioeconomic factors, the Mini-Mental State Examination, the Body Mass Index and the Mini Nutritional Assessment measurements.

## Methods

### Study design and sample selection

An observational survey was conducted on an Italian geographically representative sample of elderly population living in nursing homes. As previously described, only one local epidemiological survey on tooth loss prevalence in the Italian elderly population is available [9]. From 12,643,417 residents aged 65 or more [15], a power analysis (OpenEpi, Version 3, open source calculator) was run with a hypothesized frequency of outcome in the population of 44%+/− 5% and a confidence level of 99%, estimating a final sample size of 1493 people.

A cluster sample method was performed using each nursing home as a cluster. Twenty-three nursing homes located in the Italian five areas (North-West, North-East, Centre, South and Islands) were selected [15]; due to the high variation in the number of residents in each nursing home, the number of subjects enrolled was higher than calculated. A letter explaining the purpose of the survey and requesting the informed consent to participate was distributed by the personnel of the selected nursing homes and signed directly by subjects/caregivers; 2114 forms were distributed, 1998 forms signed and finally 1976 subjects were examined. The study protocol, conducted according to the Helsinki Declaration, was submitted to the Ethical Committee of University of Sassari (n° 298/2015).

### Methods

Subjects were examined in the nursing homes by one examiner, ad-hoc calibrated. An ad-hoc chart was prepared for collecting dental, demographic and socioeconomic factors.

The number of functional units (FUs) and the presence and type of prosthetic dental restorations was recorded. The number of FUs was defined as natural (i.e.*,* sound, restored and carious teeth) or artificial such as implant-supported, fixed (bridge pontics), and removable prostheses. Dentine lesions with extensive coronal destruction and missing teeth were recorded as non-functional, following the WHO indications [16].

From medical charts, the following data was obtained: age, gender, the Mini-Mental State Examination (MMSE), the Body Mass Index (BMI), the Mini Nutritional Assessment (MNA) and information regarding the private payment or public support of the fee of the nursing homes.

The MMSE is a 30-point questionnaire, extensively used in clinical and research settings, to measure cognitive impairment and screen for dementia [17].

The BMI is a measurement to quantify the amount of tissue mass (muscle, fat, and bone) in an individual but it is not a measurement of body fat; it is calculated by dividing a person’s weight in kilograms by the height in meters squared (kg/m^2^). It is usually categorized as underweight, normal weight, overweight or obese.

The MNA has been proposed to provide a single and easy assessment of nutritional status of elderly patients in outpatient clinics, hospitals and nursing homes [18].

### Data analysis

Data were entered in a FileMaker Pro 9.0 Runtime database and then exported to a Microsoft Excel® spread sheet. Data was analysed using Stata/SE® (Mac version 13.1). The statistical significance level was set at α = 0.05.

Subjects were stratified into three age groups: subject with less than 70 years; subjects with an age between 70 and 79 years and subjects with 80 years or more.

The presence of functional units was coded for each arch (maxillary and mandibular): less than 7 teeth for each arch were considered insufficient for chewing [19], a number of functional units between 7 and 10 teeth was considered sufficient for chewing and a greater number of teeth (> 10) was considered good for chewing [16]. The presence and type of prosthetic dental restorations was summarized as: absent of prosthesis, fixed prosthesis, removable prosthesis, combined prosthesis.

For MMSE, any score greater than or equal to 24 points out of 30 indicates a normal cognitive function; 19–23 points mild cognitive impairment, 10–18 points moderate and ≤ 9 points severe impairment [17].

BMI considers important underweight values below 17.50 kg/m^2^, underweight values between 17.51 and 19.50 kg/m^2^, ideal values between 19.51 and 23 kg/m^2^, overweight values between 23.01 and 30 kg/m^2^ and finally obesity values greater than 30 kg/m^2^.

The sum of the MNA scores distinguishes elderly patients in: 1) adequate nutritional status MNA ≥ 24; 2) at risk of malnutrition MNA between 17 and 23.5; 3) with protein-calories malnutrition MNA < 17 [18, 20].

MMSE, BMI and MNA data was not recorded homogeneously in all nursing homes, consequently, this data was not available in all enrolled sample.

The payment of the fee of the nursing homes was used as a proxy of the Socio-Economic-Status of the subjects (SES): in Italy, a subject with an average annual income over 20,000 Euros, has to pay a fee to be admitted in a nursing home.

Trend estimation was used to make statements about tendencies in the data. After stratification by age groups, the associations between oral related data and variables derived by medical chart were assessed using the chi-squared test; for values less than five, Fisher’s exact test was performed. The nonparametric test for trend across ordered groups (namely area of living, shortened functional units, etc.) was performed. A logistic model for functional units sufficient for chewing was carried out using a forward stepwise procedure estimating the ORs of functional units and the covariates. The comparison was performed using the group with the less favourable exposure as reference namely: age group (≥80 years), MMSE (Severe) and BMI (Important underweight). The Akaike Information Criterion was used to measure the goodness of fit of the statistical model. The possible modifying effects of covariates on the outcomes were tested through an interaction model (likelihood-ratio test statistic).

## Results

**Fig. 1:**
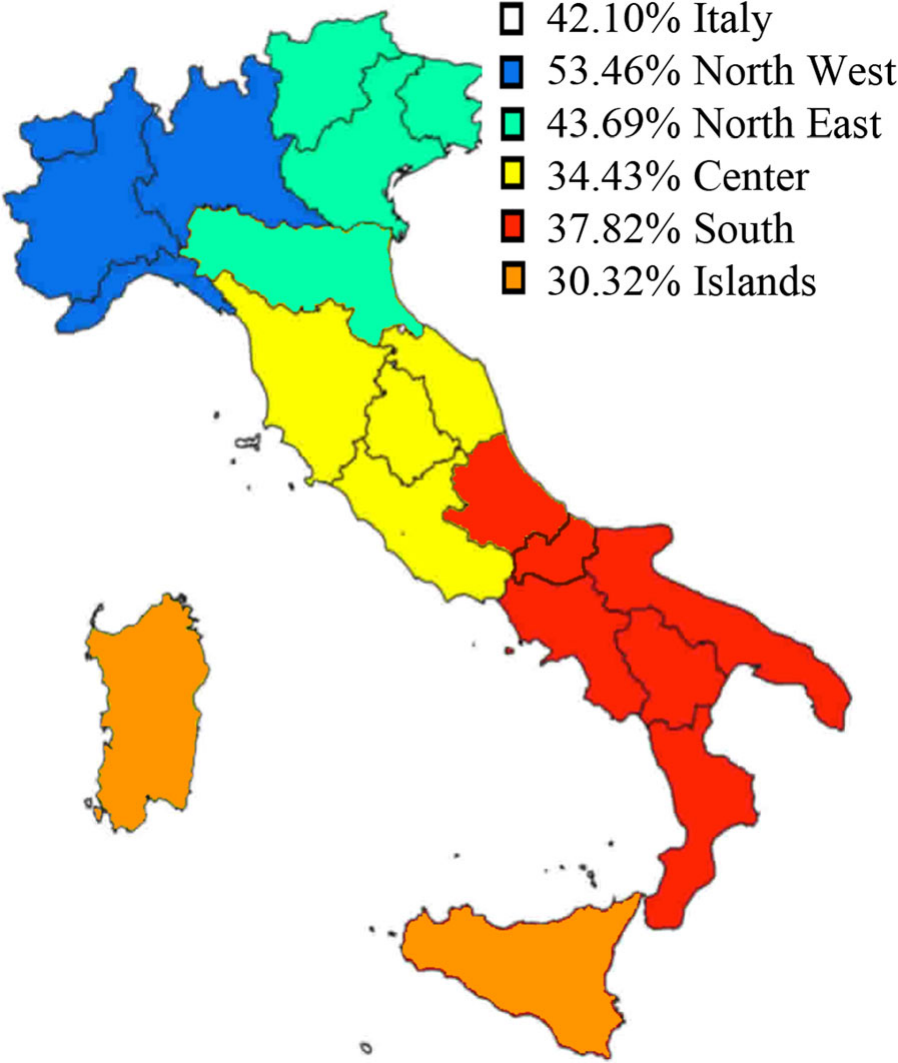
The prevalence and distribution of edentulism in the examined population across the five Italian areas

The sample mean age was 84.09 ± 9.68. Almost three quarters (74.37%) of the study sample were above 80 years old, 17.27% were 70–79 years old, and only 8.35% were less than 70 years old. A statistically significant linear trend was observed between age groups and area of living: i.e. in the oldest age group (≥80 years) the prevalence ranged from 86.89% in the Central Area to 61.96% in Islands (*data not in table*). Almost three quarters (74.04%) of the study sample were women. Globally, the prevalence of edentulism was 42.10% with a large variation among the Italian five (from 34.43% in Centre to 53.46% in North-West) (Fig. 1). Edentulism was the preeminent figure in each age group with a statistically linear trend across ages (*p* <  0.01) (*data not in table*).

MMSE, BMI and MNA were collected respectively in the 65.54%, 67.15% and 37.55% of the sample. Regardless age stratification, all considered variables, except MNA, were statistically significant associated to FUs (*p* = 0.03 for gender and MMSE and *p* <  0.01 for the others); BMI and area of living were associated to prosthetic dental restorations (*p* <  0.01) *(data not in table)*.

The sample distribution, stratified by age groups, among the presence of functional units and prosthetic dental restorations, MMSE, BMI, MNA by gender and SES is displayed in Table 1. Following age stratification, the number of functional units was statistically different regarding gender and SES in the age group 70–79 (*p* = 0.02 and *p* = 0.04, respectively). The type of prosthetic dental restorations was statistically significant associated to SES in the two oldest age groups (*p* = 0.04 and *p* < 0.01, respectively) (Table 1).

**Table 1:** Sample distribution, stratified by age groups (< 70 years, 70–79 years, ≥80 years) across gender and SES as number and percentage (%) among functional units (Upper + Lower), type of prosthetic dental restorations (Upper + Lower), MMSE, BMI and MNA

	Age group											
	< 70				70–79				≥80			
	Gender		SES		Gender		SES		Gender		SES	
	Men	Women	Low	High	Men	Women	Low	High	Men	Women	Low	High
Functional units	*p* = 0.46		*p* = 0.81		*p= 0.02*		*p= 0.04*		*p* = 0.67		*p* = 0.12	
Insufficient for chewing	64 (38.10)	55 (32.74)	28 (27.72)	44 (43.57)	104 (30.86)	176 (52.23)	92 (32.86)	147 (52.50)	245 (16.88)	1044 (71.95)	394 (30.95)	739 (58.05)
Sufficient for chewing	11 (6.54)	10 (5.95)	7 (6.93)	8 (7.92)	11 (3.27)	14 (4.15)	13 (4.64)	7 (2.50)	17 (1.17)	58 (4.00)	27 (2.12)	34 (2.67)
Good for chewing	13 (7.74)	15 (8.93)	5 (4.95)	9 (8.91)	18 (5.34)	14 (4.15)	6 (2.14)	15 (5.36)	27 (1.86)	60 (4.14)	34 (2.67)	45 (3.54)
Prosthetic dental restorations	*p* = 0.81		*p* = 0.36		*p* = 0.62		*p= 0.04*		*p* = 0.18		*p< 0.01*	
Absence	54 (33.33)	54 (33.33)	26 (26.00)	33 (33.00)	71 (21.07)	97 (28.78)	59 (21.07)	75 (26.79)	128 (8.83)	455 (31.40)	208 (16.35)	277 (21.78)
Fixed	10 (6.17)	6 (3.71)	2 (2.00)	10 (10.00)	21 (6.23)	31 (9.20)	19 (6.78)	21 (7.50)	47 (3.24)	175 (12.08)	64 (5.03)	144 (11.32)
Removable	13 (8.03)	14 (8.64)	8 (8.00)	13 (13.00)	36 (10.68)	69 (20.48)	32 (11.43)	63 (22.50)	105 (7.25)	486 (33.54)	171 (13.44)	360 (28.30)
Combined	6 (3.70)	5 (3.09)	3 (3.00)	5 (5.00)	5 (1.48)	7 (2.08)	1 (0.36)	10 (3.57)	7 (0.48)	46 (3.18)	11 (0.87)	37 (2.91)
MMSE	*p* = 0.56		*p* = 0.24		*p* = 0.07		*p* = 0.40		*p* = 0.84		*p* = 0.18	
No impairment	46 (39.32)	31 (26.49)	21 (25.93)	36 (44.44)	56 (24.45)	73 (31.88)	48 (23.64)	65 (32.02)	90 (9.54)	350 (37.12)	161 (19.05)	227 (26.86)
Suspected	4 (3.42)	3 (2.56)	3 (3,70)	2 (2.47)	5 (2.18)	11 (4.80)	10 (4.93)	5 (2.46)	6 (0.63)	39 (4.14)	15 (1.78)	27 (3.20)
Mild	6 (5.13)	4 (3,42)	5 (6.17)	3 (3,70)	11 (4.80)	10 (4.37)	8 (3.94)	11 (5.42)	22 (2.33)	85 (9.01)	42 (4.97)	53 (6.27)
Moderate	6 (5.13)	5 (4.27)	1 (1,24)	2 (2.47)	7 (3.06)	28 (12.23)	11 (5.42)	18 (8.87)	36 (3.82)	160 (16.97)	83 (9.82)	98 (11.60)
Severe	4 (3.42)	8 (6.84)	1 (1,24)	7 (8.64)	10 (4.37)	18 (7.86)	10 (4.93)	17 (8.37)	30 (3.18)	125 (13.26)	46 (5.44)	93 (11.01)
BMI	*p* = 0.08		*p* = 0.05		*p* = 0.10		*p* = 0.25		*p* = 0.23		*p* = 0.23	
Important underweight	2 (1.91)	2 (1.91)	1 (1.49)	0 (0.00)	5 (2.25)	13 (5.86)	11 (5.76)	5 (2.62)	12 (1.20)	88 (8.81)	37 (4.09)	53 (5.86)
Underweight	3 (2.86)	9 (8.57)	1 (1.49)	9 (13.43)	8 (3.60)	23 (10.36)	13 (6.81)	15 (7.85)	30 (3.00)	127 (12.71)	48 (5.30)	95 (10.50)
Ideal	6 (5.71)	9 (8.57)	3 (4.48)	9 (13.43)	24 (10.81)	31 (13.97)	19 (9.95)	31 (16.23)	60 (6.01)	239 (23.92)	98 (10.83)	181 (20.00)
Overweight	29 (27.62)	15 (14.28)	14 (20.90)	11 (16.42)	41 (18.47)	47 (21.17)	31 (16.23)	40 (20.94)	70 (7.01)	251 (25.13)	110 (12.15)	177 (19.56)
Obese	18 (17.14)	12 (11.43)	9 (13.43)	10 (14.93)	8 (3.60)	22 (9.91)	14 (7.33)	12 (6.28)	20 (2.00)	102 (10.21)	49 (5.41)	57 (6.30)
MNA	*p* = 0.42		*p* = 0.26		*p* = 0.14		*p* = 0.37		*p* = 0.56		*p* = 1.00	
High	10 (25.64)	7 (17.95)	7 (17.95)	10 (25.64)	23 (17.04)	38 (28.15)	35 (26.32)	25 (18.80)	66 (11.64)	256 (45.15)	126 (22.18)	197 (34.68)
Average	9 (23.08)	12 (30.77)	13 (33.33)	8 (20.51)	34 (25.19)	30 (22.22)	29 (21.80)	34 (25.56)	47 (8.29)	167 (29.45)	81 (14.26)	133 (23.42)
Low	1 (2.56)	–	–	1 (2.57)	6 (4.44)	4 (2.96)	6 (4.51)	4 (3.01)	4 (0.71)	27 (4.76)	18 (3.17)	13 (2.29)

χ^2^-test and Fisher exact test when a cell has a value less than 5

Area of living and BMI (Table 2) were statistically significant associated to functional units in the oldest group (*p* < 0.01 for both variables); less than 5% of subjects living in the North-East area have sufficient/good units for chewing and this finding was the highest value recorded in the examined population. BMI was also statistically significant associated in the youngest group (*p* < 0.01), while in the middle age group (71–79 years) the statistical significance was near to be reached (*p* = 0.05); overweight/obese subjects showed the highest values of FUs (7.47% of the sample).

**Table 2:** Sample distribution, stratified by age groups (< 70 years, 70–79 years, ≥80 years) as number and percentage (%) regarding the presence of functional units across living area, MMSE, BMI and MNA

	Age group								
	< 70			70–79			≥80		
	Functional units (upper + lower)								
	Insufficient for chewing	Sufficient for chewing	Good for chewing	Insufficient for chewing	Sufficient for chewing	Good for chewing	Insufficient for chewing	Sufficient for chewing	Good for chewing
AREA		*p* = 0.29			*p* = 0.74			***p*** **< 0.01**	
North West	19 (11.66)	6 (3.68)	3 (1.84)	76 (22.55)	4 (1.18)	5 (1.49)	342 (23.57)	4 (0.28)	15 (1.03)
North East	33 (20.25)	3 (1.84)	6 (3.68)	85 (25.23)	8 (2.37)	13 (3.86)	452 (31.15)	31 (2.14)	35 (2.41)
Centre	5 (3.07)	3 (1.84)	–	21 (6.23)	2 (0.59)	1 (0.30)	174 (11.99)	19 (1.31)	19 (1.31)
South	38 (23.31)	5 (3.07)	6 (3.68)	49 (14.54)	6 (1.78)	6 (1.77)	181 (12.47)	9 (0.62)	12 (0.83)
Islands	24 (14.72)	4 (2.45)	8 (4.91)	49 (14.54)	5 (1.49)	7 (2.08)	140 (9.65)	12 (0.83)	6 (0.41)
MMSE		*p* = 0.14			*p* = 0.28			*p* = 0.88	
No impairment	62 (52.99)	7 (5.98)	8 (6.84)	111 (48.47)	9 (3.93)	9 (3.93)	397 (42.10)	20 (2.12)	23 (2.44)
Suspected	4 (3.42)	3 (2.56)	–	16 (6.99)	–	–	41 (4.35)	2 (0.21)	2 (0.21)
Mild	7 (5.98)	2 (1.71)	1 (0.86)	16 (6.99)	2 (0.87)	3 (1.31)	98 (10.39)	3 (0.32)	6 (0.64)
Moderate	7 (5.98)	3 (2.56)	1 (0.86)	28 (12.23)	2 (0.87)	5 (2.18)	174 (18.45)	6 (0.64)	16 (1.69)
Severe	8 (6.84)	1 (0.86)	3 (2.56)	25 (10.92)	3 (1.31)	–	137 (14.53)	8 (0.85)	10 (1.06)
BMI		***p*** **= 0.01**			*p* = 0.05			***p*** **< 0.01**	
Important underweight	2 (1.91)	–	2 (1.91)	18 (8.11)	–	–	91 (9.11)	4 (0.40)	5 (0.50)
Underweight	6 (5.71)	3 (2.85)	3 (2.85)	22 (9.91)	3 (1.35)	6 (2.70)	144 (14.42)	4 (0.40)	9 (0.90)
Ideal	11 (10.48)	2 (1.91)	2 (1.91)	50 (22.52)	2 (0.90)	3 (1.35)	262 (26.23)	20 (2.00)	17 (1.70)
Overweight	35 (33.33)	8 (7.62)	1 (0.95)	69 (31.08)	8 (3.61)	11 (4.96)	267 (26.73)	18 (1.80)	36 (3.60)
Obese	25 (23.81)	1 (0.95)	4 (3.81)	28 (12.61)	2 (0.90)	–	112 (11.21)	9 (0.90)	1 (0.10)
MNA		*p* = 0.60			*p* = 0.71			*p* = 0.14	
Low	12 (30.77)	4 (10.26)	1 (2.56)	52 (38.52)	5 (3.70)	4 (2.96)	289 (50.97)	8 (1.41)	25 (4.41)
Average	14 (35.90)	3 (7.69)	4 (10.26)	50 (37.04)	6 (4.44)	8 (5.93)	191 (33.68)	11 (1.94)	12 (2.12)
High	1 (2.56)	–	–	9 (6.67)	1 (0.74)	–	27 (4.76)	3 (0.53)	1 (0.18)

Data captured in bold signifies statistical significance

The 96.33% of the removable prosthetic dental restorations were complete dentures. All variables, except MNA, were statistically significant associated to the type of prosthetic dental restorations in the oldest age group (≥80 years) (*p* < 0.01 for area of living and MMSE and *p* = 0.01 for BMI); the presence of any kind of prosthetic dental restorations was higher in the North-East area of Italy in all age groups (from 11.11% in younger to 11.32% in the oldest age group). Subjects with no mental impairment showed the highest percentage of prosthetic dental restorations in all age groups with a prevalence of 31.75% in the oldest group (Table 3).

**Table 3:** Sample distribution, stratified by age groups (< 70 years, 70–79 years, ≥80 years) as number and percentage (%) regarding the type of prosthetic dental restorations across living area, MMSE, BMI and MNA

	Age group											
	< 70				71–79				≥80			
	Prosthetic dental restorations											
	Absence	Fixed	Removable	Combined	Absence	Fixed	Removable	Combined	Absence	Fixed	Removable	Combined
AREA		***p*** **= 0.04**				*p* = 0.57				***p*** **< 0.01**		
North West	19 (11.73)	3 (1.85)	5 (3.09)	1 (0.62)	43 (12.76)	8 (2.37)	29 (8.61)	5 (1.48)	151 (10.42)	44 (3.04)	155 (10.70)	11 (0.76)
North East	18 (11.11)	7 (4.32)	10 (6.17)	7 (4.32)	49 (14.54)	17 (5.05)	34 (10.09)	6 (1.78)	164 (11.32)	99 (6.83)	227 (15.67)	26 (1.79)
Centre	4 (2.47)	1 (0.62)	2 (1.23)	1 (0.62)	12 (3.56)	5 (1.48)	7 (2.08)	–	80 (5.52)	47 (3.24)	77 (5.31)	8 (0.55)
South	38 (23.46)	3 (1.85)	6 (3.70)	1 (0.62)	33 (9.79)	11 (3.26)	16 (4.75)	1 (0.30)	96 (6.63)	20 (1.38)	81 (5.59)	5 (0.34)
Islands	29 (17.90)	2 (1.23)	4 (2.47)	1 (0.62)	31 (9.20)	11 (3.26)	19 (5.64)	–	92 (6.35)	12 (0.83)	51 (3.52)	3 (0.21)
MMSE		*p* = 0.06				*p* = 0.40				***p*** **< 0.01**		
No impairment	41 (35.35)	7 (6.03)	23 (19.83)	6 (5.17)	58 (25.33)	21 (9.17)	42 (18.34)	8 (3.49)	141 (14.97)	43 (4.57)	232 (24.63)	24 (2.55)
Suspected	5 (4.31)	2 (1.73)	–	–	10 (4.37)	1 (0.44)	5 (2.18)	–	15 (1.59)	4 (0.43)	24 (2.55)	2 (0.21)
Mild	5 (4.31)	3 (2.59)	–	2 (1.72)	8 (3.49)	6 (2.62)	7 (3.06)	–	38 (4.03)	19 (2.02)	46 (4.88)	3 (0.32)
Moderate	9 (7.76)	–	1 (0.86)	–	22 (9.61)	2 (0.87)	11 (4.80)	–	88 (9.34)	37 (3.93)	62 (6.58)	9 (0.96)
Severe	9 (7.76)	1 (0.86)	1 (0.86)	1 (0.86)	15 (6.55)	3 (1.31)	10 (4.37)	–	77 (8.17)	25 (2.65)	51 (5.41)	2 (0.21)
BMI		*p* = 0.27				*p* = 0.17				***p*** **= 0.01**		
Important underweight	4 (3.85)	–	–	–	7 (3.16)	1 (0.45)	9 (4.05)	1 (0.45)	42 (4.20)	22 (2.20)	36 (3.60)	–
Underweight	11 (10.58)	–	–	1 (0.96)	17 (7.66)	2 (0.90)	12 (5.41)	–	63 (6.31)	27 (2.70)	55 (5.51)	12 (1.20)
Ideal	8 (7.69)	1 (0.96)	4 (3.85)	2 (1.92)	20 (9.01)	9 (4.05)	21 (9.46)	5 (2.25)	113 (11.31)	51 (5.11)	127 (12.71)	8 (0.80)
Overweight	22 (21.16)	9 (8.65)	8 (7.69)	4 (3.85)	43 (19.37)	17 (7.66)	23 (10.36)	5 (2.25)	111 (11.11)	65 (6.51)	138 (13.81)	7 (0.70)
Obese	19 (18.27)	2 (1.92)	7 (6.73)	2 (1.92)	19 (8.56)	2 (0.90)	9 (4.05)	–	47 (4.71)	13 (1.30)	57 (5.71)	5 (0,50)
MNA		*p* = 0.18				*p* = 0.21				*p* = 0.57		
Low	22 (56.41)	3 (7.69)	10 (25.64)	3 (7.69)	67 (49.63)	18 (13.33)	34 (25.19)	7 (5.19)	214 (37.74)	95 (16.76)	208 (36.68)	21 (3.70)
Average	–	–	–	–	–	–	2 (1.48)	–	6 (1.06)	2 (0.35)	6 (1.06)	–
High	–	1 (2.57)	–	–	2 (1.48)	1 (0.74)	3 (2.22)	1 (0.74)	9 (1.59)	–	6 (1.06)	–

Data captured in bold signifies statistical significance

The outcome of the multinomial logistic regression is displayed in Table 4. The FUs category was used as dependent variable. MMSE (severe impairment), age groups (> 80 years) and gender (female) contributed significantly in the model (*p* < 0.01 for MMSE and age groups, 0.02 for gender). Subjects with a severe cognitive impairment and ≥ 80 years had an increased risk for insufficient FUs for chewing (OR = 1.19 _95%_CI 1.05–1.35 and OR = 2.07 _95%_CI 1.22–3.64, respectively); female gender showed a decreased risk (OR = 0.61 _95%_CI 0.40–0.92, respectively). The process of assessing the model allowed discovering that MNA had a modifier effect on BMI and MMSE as both indices are included in MNA. MNA showed a high number of missing data, and so it was dropped from the analysis.

**Table 4:** Multinomial logistic regression analysis. The FUs category was used as dependent variable. The comparison was performed using the less favourable level of each variable as reference

Number of observations = 1935	Log likelihood = − 459.17		*p* < 0.01
Insufficient for chewing	OR (SE)	*P* > |z|	95% Conf. Interval
MMSE (Severe)	1.19 (0.07)	**< 0.01**	1.05–1.35
BMI (Important underweight)	1.72 (0.09)	0.12	0.95–1.24
Age groups (≥80 yy)	2.07 (0.83)	**< 0.01**	1.22–3.64
Gender (female)	0.61 (0.13)	**0.02**	0.40–0.92

Data captured in bold signifies statistical significance

## Discussion

This is the first national survey reporting data on chewing ability and possible related variables in Italian elderly population. The findings show a high burden of tooth loss: an association between tooth loss and age groups, gender, area of living and income was found.

Other studies have successfully adopted the following approach to evaluate the severity of tooth loss and its associated factors [21, 22]; tooth loss was classified into three degrees of severity: a number of functional units good for chewing in each arch (ten or more), a number of functional units sufficient for chewing (between 7 and 10) and a number of units insufficient for chewing (less than 7) [19].

Age, gender, marital status, time of last dental visit, tooth type and having extraction experience were described as the best predictors for tooth loss [23]. In this survey, age has proved to be a further predisposing factor associated with insufficient number of functional units for chewing. This finding is in agreement with previous reports [22, 24]; nevertheless, the design of this survey as a cross-sectional study does not allow determining whether the association between age and number of functional units observed results from the aging process itself or results from an age cohort effect. Female gender was statistically associated to functional units for chewing, confirming previous findings [24, 25]; otherwise, gender distribution is highly skewed towards female. In developing countries, it was speculated that the low number of functional units in females might be linked to the higher prevalence of dental treatments, leading to more tooth loss [24, 26]; this association is not sufficiently documented in the Western population. Regardless of gender, one speculation might be that the increasingly common tooth extraction is due to the co-morbidity of elderly population.

Several nursing homes were selected in different geographical areas of Italy in order to provide data representative of the entire nation. The different pattern of the presence of functional units and presence and type of prosthetic dental restorations in the different geographical areas (higher in the North, lower in the Centre and South) is probably correlated with the socioeconomic/behavioural conditions of the population: people living in Northern Italy, where the social/behavioural is significantly higher than in other parts of Italy, have more access to dental care and consequently a higher level of oral health. The payment of a fee for staying in nursing homes was used as a proxy of the income and it was associated with a lower number of functional units or edentulism. In Brazil, a low socioeconomic level has been associated with an increased risk in tooth loss [26]. In Italy, dental care is only partially included in the public health service and dental examinations are usually problem-oriented, thus it is possible to speculate that subjects with fewer teeth are likely to go to the dentist only when a problem arises and usually only for extraction. The association between dental care and SES was recently described in Italian adults, in which the highest number of caries lesions was detected in subjects with the worst social/behavioural conditions [27]. However, edentulism was dominant in all geographical areas, indicating a need for dental care and a lack of control over dental diseases.

Oral health status also has an impact on physical, psychological and social well being; several studies [28, 29] have demonstrated that the elderly population has problems in chewing and eating, showing a positive association between mastication and cognitive function, including dementia. Deterioration in oral health (likewise tooth loss) increases depressive symptoms among older adults, independently from mental and physical conditions, reducing the individual well being [29]. In the present examined sample, the presence of functional units insufficient for chewing and the absence of prosthetic dental restorations were associated to MMSE index, to the BMI index, and the MNA index. Potential explanations for these associations might consider different pathways connecting tooth loss to discomfort, pain, and functional limitations, which in turn might lead to physical and mood disorders. Moreover, it was demonstrated that chewing might represent a useful approach in preserving and promoting the hippocampus-dependent cognitive function in older people [28, 30]. A significant relationship between mastication and incidence of dementia has been described and scientifically evaluated [28], suggesting that decreased chewing ability might be involved in dementia development.

Elderly people are particularly vulnerable to dietary restrictions with possible consequences on their nutritional status; having more than twenty teeth increased the likelihood of having an acceptable BMI [31]. The relationship between tooth loss and BMI has been speculated but the significance is usually biased and confounded by common risk factors that play with both conditions. In this survey, the significance of the association between BMI, remaining functional units and presence and type of prosthetic dental restorations is biased by the high number of subjects with an insufficient number of functional units (around three quarters of the sample) and absence of prosthetic dental restorations (almost half of studied population).

Participants with worse oral status (namely number of functional units insufficient for chewing and absence of prosthetic dental restorations) had lower MNA values. The hypothesis of the protective effect of a sufficient/good number of functional units against the risk of malnutrition was not proved; the absence of association may be linked to the small number of subjects classified into sufficient/good number of functional units categories and to high number of missing data, which may have resulted in a lack of statistical power [32, 33].

The main limitation of this survey is related to the enrolment modality since only elderly people living in nursing homes were included, making it impossible to generalize the results to the entire Italian population of the same age living in their own home.

One of the strengths of the present survey is the wide sample included, selected from a nationally representative sample of elderly population living in nursing homes, providing credit to the external validity of the findings.

A limitation of this survey is related to the study design: the cross-sectional nature of the investigation does not allow for the clarification of the directionality of the associations between the number of functional units and the presence of prosthetics dental restorations and the predisposing factors or the timeframe of exposure.

However, prospective studies on oral health in the elderly population are troublesome, especially in Italy, where data at national level on oral health is not available.

## Conclusions

This national survey highlights the high prevalence of edentulism and the low number of functional units in the Italian elderly population living in nursing homes, especially in oldest age groups. However, a distinctive geographical distribution of edentulism across the different areas of Italy was observed. Furthermore, the presence and the type of prosthetic dental restorations is linked to the mental conditions (MMSE): the higher is the mental impairment the higher is the number of subjects with absence of prosthetic restorations.

The findings of this national survey highlight the need for public health policy, aiming to increase awareness regarding oral health though health education.

## Additional file

Additional file 1: Where you list the following information for each additional/supplementary file in the file inventory: File name raw data.xlsx, The file includes all the raw data of the survey, The data are in a excel file. (XLSX 326 kb)

## Abbreviations

BMI: Body Mass Index
FUs: functional units
MMSE: Mini-Mental State Examination
MNA: Mini Nutritional Assessment
